# Signal peptide peptidase (SPP) dimer formation as assessed by fluorescence lifetime imaging microscopy (FLIM) in intact cells

**DOI:** 10.1186/1750-1326-1-16

**Published:** 2006-11-14

**Authors:** Andrew C Nyborg, Lauren Herl, Oksana Berezovska, Anne V Thomas, Thomas B Ladd, Karen Jansen, Bradley T Hyman, Todd E Golde

**Affiliations:** 1 Department of Neuroscience, Mayo Clinic Jacksonville, Mayo Clinic College of Medicine, Jacksonville, Florida 32224, USA; 2 Alzheimer's Disease Research Unit, Massachusetts Institute for Neurodegenerative Diseases, Massachusetts General Hospital, Charlestown, Massachusetts 02129, USA

## Abstract

**Background:**

Signal peptide peptidase (SPP) is an intramembrane cleaving protease identified by its cleavage of several type II membrane signal peptides. Conservation of intramembrane active site residues demonstrates that SPP, SPP family members, and presenilins (PSs) make up a family of intramembrane cleaving proteases. Because SPP appears to function without additional protein cofactors, the study of SPP may provide structural insights into the mechanism of intramembrane proteolysis by this biomedically important family of proteins. Previous studies have shown that SPP isolated from cells appears to be a homodimer, but some evidence exists that in vitro SPP may be active as a monomer. We have conducted additional experiments to determine if SPP exists as a monomer or dimer *in vivo*.

**Results:**

Fluorescence lifetime imaging microscopy (FLIM) can be is used to determine intra- or intermolecular interactions by fluorescently labeling epitopes on one or two different molecules. If the donor and acceptor fluorophores are less than 10 nm apart, the donor fluorophore lifetime shortens proportionally to the distance between the fluorophores. In this study, we used two types of fluorescence energy transfer (FRET) pairs; cyan fluorescent protein (CFP) with yellow fluorescent protein (YFP) or Alexa 488 with Cy3 to differentially label the NH2- or COOH-termini of SPP molecules. A cell based SPP activity assay was used to show that all tagged SPP proteins are proteolytically active. Using FLIM we were able to show that the donor fluorophore lifetime of the CFP tagged SPP construct in living cells significantly decreases when either a NH2- or COOH-terminally YFP tagged SPP construct is co-transfected, indicating close proximity between two different SPP molecules. These data were then confirmed in cell lines stably co-expressing V5- and FLAG-tagged SPP constructs.

**Conclusion:**

Our FLIM data strongly suggest dimer formation between two separate SPP proteins. Although the tagged SPP constructs are expressed throughout the cell, SPP dimer detection by FLIM is seen predominantly at or near the plasma membrane.

## Background

Signal peptide peptidase (SPP) is a member of a larger group of intramembrane cleaving proteases (I-CLiP) that play a variety of important roles in cell signaling [1] and regulation [2], cell surveillance [3], intracellular communication [4], Alzheimer's disease [5], cancer [6,7], and hepatitis C virus [8]. Within the I-CLiP family, presenilin (PS) 1 and 2, SPP, SPPL3, and SPPL2b are putative aspartyl proteases that cleave a variety of transmembrane substrates [9-14]. PSs, SPP, and SPPLs all contain a conserved active site motif of YD and GXGD in adjacent transmembrane domains [15-17]. Additionally, they contain a conserved PAL motif near the COOH-terminus that has been shown to be critical for activity [18]. Although the active sites of PSs and SPP appear to be conserved, the proteins differ in that PSs cleave type I membrane proteins and SPP cleaves type II membrane proteins [15,16,19-21]. This difference is thought to be due to the inverted active site topologies of SPP and PS [11,22,23]. In addition to the orientation difference between PS and SPP, PSs require three additional proteins to function as γ-secretase [24-29], whereas SPP appears to be capable of functioning alone [11,22,30].

SPP was originally identified as a ~45 kDa N-linked glycoprotein using an inhibitor labeling approach [11]. Other reports of SPP describe two bands, one at ~42 kDa, and one at ~95 kDa [10,30]. Co-purification of two different epitope tagged forms of SPP from a stably transfected cell line expressing both tagged versions demonstrates that the ~95 kDa band is a homodimer [30], however, unequivocal evidence of SPP dimerization in intact cells has been lacking. There is evidence that PS1 may exists as a dimer as well [31-36]. Yeast two-hybrid studies show that NH2 and COOH-terminal fragments of PS1 or intact PS1 can self-associate [32,35]. Although minor, high molecular weight forms of PS have been detected by native Western blotting after denaturing SDS-PAGE [32,35]. PS1 NTF-NTF and CTF-NTF dimers were detected following labeling with a transition state analog γ-secretase inhibitor [33]. Finally, we have shown that PS1 forms dimers using both co-immunoprecipitation and fluorescent lifetime imaging microscopy (FLIM) in intact cells [36].

Although the I-CLiP family members play a variety of important roles in biology and disease processes, a paucity of structural information exists due to the complexity of studying the multipass membrane proteins that make up this family. To date, multipass membrane proteins have proven refractory to many of the current methods used for obtaining protein structures, thus little insight into the *in vivo *nature of the active proteases exists. We set out to show that the SPP homodimer exists in intact cells using a fluorescence resonance energy transfer (FRET) based technique, FLIM. Our data demonstrate that the NH2-terminus of one SPP molecule is less than 10 nm apart from the NH2-terminus or COOH-terminus of another SPP molecule, indicating dimer formation. The SPP dimerization is demonstrated both in intact, living cells using SPP fluorescent fusion protein constructs, and in fixed intact cells using tagged SPP constructs. Both the tagged SPP constructs and the fluorescent fusion protein constructs used in the FLIM studies are active in a cell based SPP reporter activity assay [22]. These data suggest that the SPP homodimer observed on western blots at ~95 kDa is present in intact cells.

## Results

### Epitope tagged SPP constructs and CFP/YFP SPP fusion constructs maintain SPP activity

**Figure 1:**
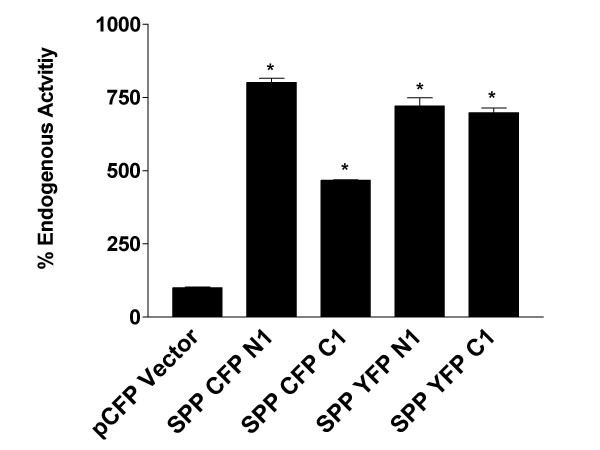
**CFP/YFP SPP fusion proteins are active in a cell based SPP reporter assay**. SPP fluorescent fusion expression plasmid or vector control was transfected into HEK 293T cells as described previously [19]. Activity is obtained by measuring the firefly luciferase activity and dividing it by the Renilla luciferase activity (transfection control). Activity of each SPP fluorescent fusion protein is plotted as % of endogenous activity. All four different SPP fluorescent fusion protein constructs significantly increase SPP reporter assay activity 5–8 fold above endogenous SPP activity. *p < 0.05

To ensure that all constructs used for FRET measurements and FLIM studies were active, a cell based SPP activity assay was used to assess SPP activity. This assay utilizes a substrate consisting of the NH2-terminus of the ATF6 transcription factor fused to a transmembrane domain susceptible to SPP cleavage in vitro [12,22,37]. Cleavage of the substrate releases soluble ATF6 from the membrane, which then translocates to the nucleus and activates an ATF6 luciferase reporter construct. Using this assay, we have previously shown that SPPNTFLAG and SPPCTV5 are both active and that overexpression of each gives a significant increase in SPP activity [22]. As shown in Figure 1, all SPP CFP- and YFP-fusion protein were catalytically active as well.

### FLIM demonstrates close proximity of two differentially tagged SPP constructs

**Figure 2:**
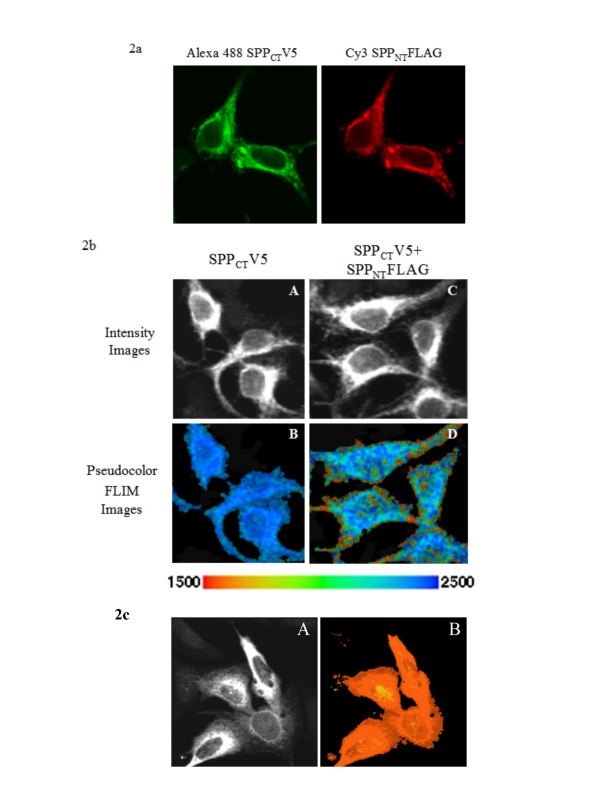
**FLIM demonstrates that tagged SPP constructs form dimers in intact cells**. **a)**. Confocal microscopy images of Alexa 488 labeled SPP_CT_V5 and Cy3 labeled SPP_NT_FLAG in stably transfected HEK cells demonstrate the cellular distribution of SPP. **b)**. FLIM was then used to monitor the proximity of Alexa 488 labeled SPP_CT_V5 and Cy3 labeled SPP_NT_FLAG molecules. Intensity images (A,C) again show the immunostaining of Alexa 488 labeled SPP_CT_V5 (donor fluorophore). The corresponding FLIM images provide the visualization of the degree of donor fluorophore lifetime shortening on a pseudocolor scale (B,D). B) Alexa 488 lifetime distribution in the absence of an acceptor fluorophore (negative control). D) The red pixels represent shortening of the donor fluorophore lifetime indicating that two SPP molecules form a dimer and come into closest proximity at the periphery of the cell. **c) **Confocal microscopy images of cells stably expressing SPP_CT_V5 were labeled by goat anti-V5 Alexa 488 and Cy3 anti-goat IgG and demonstrate that the two fluorophores are in close proximity of one another (positive control). A) The intensity image of SPP_CT_V5 Alexa 488 immunoreactivity. B) The pseudocolored FLIM image shows shortened Alexa 488 lifetime (~1300 psec, red pixels) ubiquitously distributed throughout the cell.

Confocal microscopy of epitope tagged SPP constructs stably transfected into HEK cells or SPP fluorescent fusion proteins transiently transfected into CHO cells demonstrates that SPP is uniformly distributed throughout the cell with the exception of the nucleus (Figure 2A). Previously, SPP was shown to co-localize with the ER marker BiP [38]. Although confocal microcopy shows equivalent compartment localization of all epitope tagged SPP and SPP fusion constructs employed in this study, this colocalization does not necessarily imply that there are close intermolecular interactions equally throughout the cell.

To asses the *in vivo *SPP association, a FLIM assay measuring the proximity between two differentially tagged SPP molecules was established. In this assay, the donor fluorophore lifetime is measured, which varies based on the surrounding microenvironment and is shortened in the presence of a FRET acceptor fluorophore in the immediate vicinity (<10 nm). This decrease in donor fluorophore lifetime is proportional to the distance between the donor and the acceptor fluorophores and can be visualized using a pseudocolor scale. On this pseudocolor scale, blue pixels indicate non FRETing molecules with an unchanged donor fluorophore lifetime and red pixels indicate FRETing molecules with a shortened donor fluorophore lifetime (Figure 2B and 2C).

In the live cell FLIM analysis, first the donor fluorophore (CFP) lifetime was determined in the absence of an acceptor fluorophore (~2200 psec lifetime) (Table 1). Once SPP-YFP fusion protein constructs were cotransfected together with the SPP-CFP constructs into cells, a significant decrease in CFP lifetime was observed, strongly indicating dimer formation between two different SPP molecules. This decrease in donor fluorophore lifetime was consistently observed independent from the fusion site of CFP or YFP to the NH2 or COOH-terminus of SPP, respectively (Table 1).

**Table 1:** FLIM assay detects SPP dimer formation of fusion proteins.

Donor (CFP)	Acceptor (YFP)	CFP lifetime (mean ± st.err, psec)	P value (Compared to CFP donor only)
NH2-terminus (n = 26)	empty vector	2267 ± 60	-
NH2-terminus (n = 22)	NH2-terminus	1757 ± 125	< 0.001
COOH-terminus (n = 20)	COOH-terminus	1494 ± 145	< 0.001
NH2-terminus (n = 6)	COOH-terminus	1526 ± 210	< 0.001

SPP dimer formation between all combinations of fusion protein was demonstrated by significant shortening of donor lifetime. CHO cells co-transfected with different combinations of NH2- or COOH-labeled SPP fusion protein constructs. All CFP-SPP donor lifetimes were significantly shortened in the presence of another YFP-SPP molecule. ANOVA was performed using a Dunnet's post-hoc t-test.

An HEK cell line coexpressing both COOH-terminal V5 tagged SPP (SPP_CT_V5) and a NH2-terminal FLAG tagged SPP (SPP_NT_FLAG) was previously generated and used to isolate an *in vitro *dimer of SPP [30]. In these experiments, a cell line expressing only SPP_CT_V5, which was tagged with Alexa 488 as a donor fluorophore, was used as a negative control. Alexa 488 lifetime was ~2500 psec. (Table 2). In the SPP_NT_FLAG/SPP_CT_V5 co-expressing cell line a significant decrease in donor fluorophore lifetime was observed relative to the negative control (~2200 psec lifetime) (Table 2), again indicating close proximity between two different SPP molecules. Although SPP is present throughout most of the cell, the majority of SPP dimers form at or near the plasma membrane, as the most pronounced decrease in donor fluorophore lifetime is detected in this region (red pixels) (Figure 2B).

**Table 2:** FLIM assay detects SPP dimer formation of epitope tagged SPP constructs.

Donor (Alexa 488 labeled)	Acceptor (Cy3 labeled)	Alexa 488 Lifetime (mean ± st.err, psec)	P value (Compared to Alexa donor only)
SPP_CT_V5* (n = 18)	None	2504 ± 84	-
SPP_CT_V5* (n = 18)	SPP_NT_FLAG**	1978 ± 66	<0.001
SPP_CT_V5* (n = 5)	Anti-goat IgG	1328 ± 58	<0.001

*Goat Anti-V5

**Mouse Anti-FLAG

SPP dimer formation between all combinations of epitope tagged SPP constructs was demonstrated by significant shortening of donor lifetime. HEK 293 cells stably overexpressing SPP_CT_V5 and SPP_NT_FLAG. Alexa 488 labeled SPP_CT_V5 donor fluorophore lifetime was significantly shortened in the presence of either Cy3 labeled SPP_NT_FLAG or Cy3 labeled anti-goat IgG (positive control). ANOVA was performed using a Dunnet's post-hoc t-test.

## Discussion

Under mild lysis conditions, SDS-PAGE, and Western blotting SPP is primarily detected as a ~95 kDa homodimer [30]. The SDS-stable homodimer is dissociable to a monomer by heating in the presence of SDS and reductant [30]. In addition, we have shown that a NH2-terminally FLAG epitope tagged SPP co-purifies with a COOH-terminally V5 his epitope tagged SPP construct [30]. These biochemical studies suggest that SPP is likely to exist as a dimer *in vivo*. However, *in vitro *studies demonstrate that detergent solubilized monomeric SPP is capable of cleaving exogenous synthetic peptide substrates [39]. To determine if the SPP dimer is present in intact cells, a FLIM assay was established to measure the proximity between two different SPP molecules in live and fixed cells. FLIM has been described as a novel technique for the analysis of protein proximity [40,41] and was used to show that PS1 interacts with amyloid precursor protein and low density receptor related protein in the same compartment making one a competitive inhibitor of the other [42]. Association of two SPP constructs was shown by the fact that the donor fluorophore lifetime of a CFP or Alexa 488 labeled SPP construct significantly decreases once an acceptor fluorophore of an YFP or Cy3 labeled SPP construct is present in the cell, indicating close proximity of two different SPP molecules. SPP dimer formation was seen between two differentially labeled NH2-termini and two differentially labeled COOH-termini with CFP- and YFP-fusion constructs in live cells. Dimer formation was also seen between the NH2-terminus of one SPP molecule and the COOH-terminus of another SPP molecule using both fusion proteins and epitope tags (Table 1 and 2). These data indicate that the epitope tags of two differentially tagged SPP constructs are less than 10 nm apart.

Both confocal (Figure 2A) and intensity images (Figure 2B) show that the tagged SPP constructs are expressed throughout the cell, excluding the nucleus. FLIM results provide strong evidence for a close association of two SPP molecules at or near the plasma membrane. Based on previous work it is generally accepted that SPP is predominantly localized to the endoplasmic reticulum (ER) [14,23,38,43-46]. As known substrates of SPP are also found in the ER, it is hypothesized that SPP functions proteolytically within the ER [8,43,47,48]. SPP contains a putative COOH-terminal KKXX ER retention signal that does not appear to always retain SPP in the ER and is also not required for activity [22,30,44]. In fact, epitope tags placed after the KKXX ER retention signal do not to effect dimerization or activity [22,30,44]. Not all naturally occurring SPP constructs are confined to the ER [45]. Recently, a splice variant of SPP (SPPβ) was shown to be located primarily at the plasma membrane [45]. Our biochemical data would suggest that the majority of SPP exists in a dimeric form and the FLIM data provide evidence for a closely associated homodimer at or near the plasma membrane, but not in the ER. Whether this represents a difference in the SPP association in various subcellular locations or a limitation of these techniques is not clear at the present time.

All constructs used in these experiments are active in cell based SPP reporter assays (Figure 1). We see no difference between COOH-terminal, NH2-terminal, or untagged SPP with regard to their ability to cleave a cell based substrate or form a dimer (Figure 1) [22,30]. Although our biochemical studies suggest that SPP exists primarily as a dimer, the FLIM data support this finding at or neat the plasma membrane. None of these studies provide insight into whether SPP functions as a dimer or monomer or where activity is localized. We believe that this issue will take some time to resolve. Indeed, it took some time to resolve the spatial activity paradox observed with presenilins where the majority of presenilin is localized in the ER, whereas γ-secretase activity largely resides in other cellular compartments (late trans-golgi, plasma membrane, endosomes) [49]. Thus it is possible that a relatively small SPP pool contributes to the majority of the activity observed.

SPP is a multipass membrane protein containing seven transmembrane domains with the NH2-terminus in the lumen (extracellular) and the COOH-terminus in the cytosol [15,23]. We interpret the FRET experiment to show interactions between two closely associated molecules even though the fluorophores are on opposite ends of each molecule. While detecting FRET across a membrane is not always possible due to the distances involved, it can be accomplished if there are favorable alignments [50].

Though detailed structural data for PS, SPP and other I-CLiPs do not yet exist, it is clear that some portion of cellular SPP and PS are present as dimers [32,33,35,36]. SPP is apparently a simpler aspartyl I-CLiP than γ-secretase in that: SPP can be expressed in active forms without additional co-factors in heterologous systems, and, unlike PS, SPP does not appear to undergo or require endoproteolysis for activity [51]. Thus, SPP may be expected to be more easily characterized at a molecular and structural level than γ-secretase. Structural information and studies will inevitably provide us with greater insight into this biologically important family of proteins and aid in developing compounds to target their activities.

## Materials and methods

### DNA constructs and cell culture

Epitope tagged SPP constructs used in the FLIM studies were described previously [30]. Cyan fluorescent protein and yellow fluorescent protein SPP fusion constructs were generated by PCR based cloning techniques using four vectors. pCFP-N1 and pYFP-N1 fuses the fluorescent protein to the COOH-terminus and pCFP-C1 and pYFP-C1 fuses the fluorescent protein to the NH2-terminus of SPP. All clones were verified by sequencing. SPP reporter assay clones were described previously [22]. Human Embryonic Kidney (HEK) 293T cells stably overexpressing COOH-terminal V5 tagged SPP (SPP_CT_V5) and NH2-terminal FLAG tagged SPP (SPP_NT_FLAG) constructs [27] were grown in Opti-MEM^© ^media (Gibco) containing 10% FBS in an incubator at 37°C supplemented with 5% CO_2_.

### SPP reporter assay

The luciferase reporter assays were performed as described previously [22]. In short, HEK 293T cells were grown to 70% confluentcy and transiently transfected using 100 μl serum free Opti MEM^® ^(Gibco), 8 μl of fugene, 0.01 μg of pRL-SV40 *Renilla *expression plasmid (Promega), 0.25 μg of pGL3 5xATF6 reporter plasmid, 0.25 μg of pAG3 SPP_sub _plasmid and 0.5 μg of the SPP construct expression plasmid or control plasmid to total 1 μg of DNA in each well of a 12 well plate. Cells were incubated with the transfection reagent for 6–12 hours, after which the serum deficient media was replaced with Dulbecco's modified Eagle's supplemented with 8% normal calf serum (Cambrex), 2% fetal bovine serum (Hyclone) and incubated for an additional 8–24 hours. Firefly and *Renilla *luciferase activities were measured using the Dual-Luciferase^® ^kit (Promega) and a Veritas microplate Luminometer (Turner Biosystems) with Veritas 2.0.40 software package. Transfections were performed in triplicate. Results were normalized to the *Renilla *luciferase activity control.

### Transient transfections

Chinese hamster ovary (CHO) cells were split into 35 mm dishes and co-transfected with CFP- and YFP-SPP fusion constructs using Superfect Transfection Reagent (Qiagen, Valencia, CA) according to the manufacturer's instructions. 24 hours post-transfection media was exchanged with Hank's Balanced Salt Solution (Gibco) immediately before live cell FLIM imaging.

### Immunocytochemistry

HEK cells stably overexpressing SPP_CT_V5 and SPP_NT_FLAGconstructs were split into four-well chamber slides 24 hours prior to immunocytochemistry and allowed to grow to confluentcy. Once confluent, the cells were fixed with 4% paraformaldehyde for 10 minutes and permeabilized in 0.1% Triton X-100 in 1.5% NDS for one hour. Primary antibodies goat anti-V5 (1:300) (Abcam, Cambridge, MA) and mouse anti-FLAG (1:600) (Sigma-Aldrich, St. Louis, MO) were applied for one hour at room temperature, followed by three washes in 1× TBS. The primary antibodies were labeled with secondary antibodies conjugated to either Alexa 488 (Invitrogen, Gaithersburg, MD) or Cy3 (Jackson Immunoresearch, West Grove, PA) for one hour at room temperature, followed by three washes in 1× TBS. Prior to FLIM analysis, slides were coverslipped using GVA Mounting Solution (Zymed, South San Francisco, CA).

### FLIM assay

FLIM has been described as a novel FRET-based technique that allows for the analysis of proximity between epitopes of one or two different molecules [40,42,52-54]. The technique is based on the observation that the fluorescence lifetime of a donor fluorophore (Alexa 488 or CFP) shortens in the presence of a acceptor fluorophore (Cy3 or YFP) in close proximity (<10 nm). The decrease in lifetime is proportional to the distance between the fluorophores at R^6^.

In these experiments, negative controls consist of cells transfected or immunostained with the donor fluorophore only. Whereas in positive controls (in the immunostained cells only), the cells are stained with the donor fluorophore, which is then labeled with a secondary fluorophore against the species in which the donor fluorophore is raised [40]. In the experimental conditions, the two epitopes of interest are labeled with donor and acceptor fluorophore, respectively.

A Radiance 2000 microscope (Bio-Rad, Hercules, CA) using a mode-locked femtosecond-pulsed Ti:Sapphire Laser (Mai Tai; Sprecta-Physics, Mountain View, CA) at 800 nm was used for multiphoton fluorescence excitation. Fluorescence lifetimes were recorded employing a high-speed photomultiplier tube (MCP R3809; Hamamatsu, Hamamatsu City, Japan) and a fast-time correlated single-photon counting acquisition board (SPC 830; Becker & Hickl, Berlin, Germany).

To determine the fraction of fluorophores within each pixel that interacts with an acceptor, donor fluorophore lifetimes were determined by fitting the FLIM data to single (negative control) or bi (positive control, experimental conditions) exponential decay curves, respectively. The display of lifetimes on a pseudocolor scale was made possible by creating a 128 × 128 pixel matrix for both single- and bi-exponential curve fit data for each pixel (SPCImage Becker & Hickl, Berlin, Germany).

### Data analysis

Data were analyzed using Sigma Stat 3.1 from Systat Software Inc. For comparison of multiple experimental values relative to controls an ANOVA was performed using a Dunnet's post hoc t-test. Variance is reported as the standard error of the mean for the SPP reporter assay and as standard deviation for the FLIM assay.

## Abbreviations

FLIM, fluorescence lifetime imaging microscopy; SPP, signal peptide peptidase; CTF, COOH-terminal fragment; FRET, fluorescence resonance energy transfer; YFP, yellow fluorescent protein; CFP, cyan fluorescent protein; I-CLiP, Intra-membrane cleaving protease; PS, presenilin; SPP_CT_V5, COOH-terminal V5 tagged SPP; SPP_NT_FLAG, NH2-terminal FLAG tagged SPP; HEK, Human Embryonic Kidney; CHO, Chinese hamster ovary.

## Authors' contributions

ACN, LH, OB, BTH and TEG contributed to the conception, design, analysis and interpretation of the data and were responsible to the manuscript preparation. ACN, LH, KJ, TBL, OB, and AVT carried out the experiments in this manuscript. ACN, LH, OB, BTH, and TEG all contributed to the interpretation and analysis of data. All authors read and approved the final manuscript.

## References

[B1] Brown MS, Ye J, Rawson RB, Goldstein JL (2000). Regulated intramembrane proteolysis: a control mechanism conserved from bacteria to humans. Cell.

[B2] Mumm JS, Kopan R (2000). Notch Signaling: From the Outside In. Dev Biol.

[B3] Lemberg MK, Bland FA, Weihofen A, Braud VM, Martoglio B (2001). Intramembrane proteolysis of signal peptides: an essential step in the generation of HLA-E epitopes. J Immunol.

[B4] Urban S, Lee JR, Freeman M (2001). Drosophila rhomboid-1 defines a family of putative intramembrane serine proteases. Cell.

[B5] Selkoe DJ (2001). Alzheimer's disease: genes, proteins, and therapy. Physiol Rev.

[B6] Sun Y, Lowther W, Kato K, Bianco C, Kenney N, Strizzi L, Raafat D, Hirota M, Khan NI, Bargo S, Jones B, Salomon D, Callahan R (2005). Notch4 intracellular domain binding to Smad3 and inhibition of the TGF-beta signaling. Oncogene.

[B7] van Es JH, van Gijn ME, Riccio O, van den Born M, Vooijs M, Begthel H, Cozijnsen M, Robine S, Winton DJ, Radtke F, Clevers H (2005). Notch/gamma-secretase inhibition turns proliferative cells in intestinal crypts and adenomas into goblet cells. Nature.

[B8] Okamoto K, Moriishi K, Miyamura T, Matsuura Y (2004). Intramembrane proteolysis and endoplasmic reticulum retention of hepatitis C virus core protein. J Virol.

[B9] Ponting CP, Hutton M, Nyborg A, Baker M, Jansen K, Golde TE (2002). Identification of a novel family of presenilin homologues. Hum Mol Genet.

[B10] Grigorenko AP, Moliaka YK, Korovaitseva GI, Rogaev EI (2002). Novel class of polytopic proteins with domains associated with putative protease activity. Biochemistry (Mosc).

[B11] Weihofen A, Binns K, Lemberg MK, Ashman K, Martoglio B (2002). Identification of signal peptide peptidase, a presenilin-type aspartic protease. Science.

[B12] Nyborg AC, Ladd TB, Jansen K, Kukar T, Golde TE (2006). Intramembrane proteolytic cleavage by human signal peptide peptidase like 3 and malaria signal peptide peptidase. Faseb J.

[B13] Fluhrer R, Grammer G, Israel L, Condron MM, Haffner C, Friedmann E, Bohland C, Imhof A, Martoglio B, Teplow DB, Haass C (2006). A gamma-secretase-like intramembrane cleavage of TNFalpha by the GxGD aspartyl protease SPPL2b. Nat Cell Biol.

[B14] Friedmann E, Hauben E, Maylandt K, Schleeger S, Vreugde S, Lichtenthaler SF, Kuhn PH, Stauffer D, Rovelli G, Martoglio B (2006). SPPL2a and SPPL2b promote intramembrane proteolysis of TNFalpha in activated dendritic cells to trigger IL-12 production. Nat Cell Biol.

[B15] Martoglio B, Golde TE (2003). Intramembrane-cleaving aspartic proteases and disease: presenilins, signal peptide peptidase and their homologs. Hum Mol Genet.

[B16] Xia W, Wolfe MS (2003). Intramembrane proteolysis by presenilin and presenilin-like proteases. J Cell Sci.

[B17] Weihofen A, Lemberg MK, Friedmann E, Rueeger H, Schmitz A, Paganetti P, Rovelli G, Martoglio B (2003). Targeting presenilin-type aspartic protease signal peptide peptidase with gamma-secretase inhibitors. J Biol Chem.

[B18] Wang J, Brunkan AL, Hecimovic S, Walker E, Goate A (2004). Conserved "PAL" sequence in presenilins is essential for gamma-secretase activity, but not required for formation or stabilization of gamma-secretase complexes. Neurobiol Dis.

[B19] Golde TE, Eckman CB (2003). Physiologic and pathologic events mediated by intramembranous and juxtamembranous proteolysis. Sci STKE.

[B20] Lemberg MK, Martoglio B (2004). On the mechanism of SPP-catalysed intramembrane proteolysis; conformational control of peptide bond hydrolysis in the plane of the membrane. FEBS Lett.

[B21] Martoglio B (2003). Intramembrane proteolysis and post-targeting functions of signal peptides. Biochem Soc Trans.

[B22] Nyborg AC, Jansen K, Ladd TB, Fauq A, Golde TE (2004). A signal peptide peptidase (SPP) reporter activity assay based on the cleavage of type II membrane protein substrates provides further evidence for an inverted orientation of the SPP active site relative to presenilin. J Biol Chem.

[B23] Friedmann E, Lemberg MK, Weihofen A, Dev KK, Dengler U, Rovelli G, Martoglio B (2004). Consensus analysis of signal peptide peptidase and homologous human aspartic proteases reveals opposite topology of catalytic domains compared with presenilins. J Biol Chem.

[B24] Yu G, Nishimura M, Arawaka S, Levitan D, Zhang L, Tandon A, Song YQ, Rogaeva E, Chen F, Kawarai T, Supala A, Levesque L, Yu H, Yang DS, Holmes E, Milman P, Liang Y, Zhang DM, Xu DH, Sato C, Rogaev E, Smith M, Janus C, Zhang Y, Aebersold R, Farrer LS, Sorbi S, Bruni A, Fraser P, St George-Hyslop P (2000). Nicastrin modulates presenilin-mediated notch/glp-1 signal transduction and betaAPP processing [see comments]. Nature.

[B25] Francis R, McGrath G, Zhang J, Ruddy DA, Sym M, Apfeld J, Nicoll M, Maxwell M, Hai B, Ellis MC, Parks AL, Xu W, Li J, Gurney M, Myers RL, Himes CS, Hiebsch R, Ruble C, Nye JS, Curtis D (2002). aph-1 and pen-2 are required for Notch pathway signaling, gamma- secretase cleavage of betaAPP, and presenilin protein accumulation. Dev Cell.

[B26] Edbauer D, Winkler E, Regula JT, Pesold B, Steiner H, Haass C (2003). Reconstitution of gamma-secretase activity. Nat Cell Biol.

[B27] Takasugi N, Tomita T, Hayashi I, Tsuruoka M, Niimura M, Takahashi Y, Thinakaran G, Iwatsubo T (2003). The role of presenilin cofactors in the gamma-secretase complex. Nature.

[B28] Kimberly WT, LaVoie MJ, Ostaszewski BL, Ye W, Wolfe MS, Selkoe DJ (2003). Gamma-secretase is a membrane protein complex comprised of presenilin, nicastrin, Aph-1, and Pen-2. Proc Natl Acad Sci U S A.

[B29] Marlow L, Canet RM, Haugabook SJ, Hardy JA, Lahiri DK, Sambamurti K (2003). APH1, PEN2, and Nicastrin increase Abeta levels and gamma-secretase activity. Biochem Biophys Res Commun.

[B30] Nyborg AC, Kornilova AY, Jansen K, Ladd TB, Wolfe MS, Golde TE (2004). Signal peptide peptidase forms a homodimer that is labeled by an active site-directed gamma-secretase inhibitor. J Biol Chem.

[B31] Cervantes S, Saura CA, Pomares E, Gonzalez-Duarte R, Marfany G (2004). Functional implications of the presenilin dimerization: reconstitution of gamma-secretase activity by assembly of a catalytic site at the dimer interface of two catalytically inactive presenilins. J Biol Chem.

[B32] Cervantes S, Gonzalez-Duarte R, Marfany G (2001). Homodimerization of presenilin N-terminal fragments is affected by mutations linked to Alzheimer's disease. FEBS Lett.

[B33] Schroeter EH, Ilagan MX, Brunkan AL, Hecimovic S, Li YM, Xu M, Lewis HD, Saxena MT, De Strooper B, Coonrod A, Tomita T, Iwatsubo T, Moore CL, Goate A, Wolfe MS, Shearman M, Kopan R (2003). A presenilin dimer at the core of the gamma-secretase enzyme: insights from parallel analysis of Notch 1 and APP proteolysis. Proc Natl Acad Sci U S A.

[B34] Hebert SS, Godin C, Levesque G (2003). Oligomerization of human presenilin-1 fragments. FEBS Lett.

[B35] Hebert SS, Godin C, Tomiyama T, Mori H, Levesque G (2003). Dimerization of presenilin-1 in vivo: suggestion of novel regulatory mechanisms leading to higher order complexes. Biochem Biophys Res Commun.

[B36] Herl L, Lleo A, Thomas AV, Nyborg AC, Jansen K, Golde TE, Hyman BT, Berezovska O (2006). Detection of presenilin-1 homodimer formation in intact cells using fluorescent lifetime imaging microscopy. Biochem Biophys Res Commun.

[B37] Wang J, Beher D, Nyborg AC, Shearman MS, Golde TE, Goate A (2006). C-terminal PAL motif of presenilin and presenilin homologues required for normal active site conformation. J Neurochem.

[B38] Krawitz P, Haffner C, Fluhrer R, Steiner H, Schmid B, Haass C (2005). Differential localization and identification of a critical aspartate suggest non-redundant proteolytic functions of the presenlin in homologues SPPL2b and SPPL3. J Biol Chem.

[B39] Sato T, Nyborg AC, Iwata N, Diehl TS, Saido TC, Golde TE, Wolfe MS (2006). Signal peptide peptidase: biochemical properties and modulation by nonsteroidal antiinflammatory drugs. Biochemistry.

[B40] Berezovska O, Randya P, Skoch J, Wolfe MS, Bacskai B, Hyman BT (2003). Amyloid precursor protein associates with a nicastrin-dependent docking site on the presenilin 1-gamma-secretase complex in cells demonstrated by fluorescence lifetime imaging. J Neurosci.

[B41] Bacskai BJ, Skoch J, Hickey GA, Allen R, Hyman BT (2003). Fluorescence resonance energy transfer determinations using multiphoton fluorescence lifetime imaging microscopy to characterize amyloid-beta plaques. J Biomed Opt.

[B42] Lleo A, Waldron E, von Arnim CA, Herl L, Tangredi MM, Peltan ID, Strickland DK, Koo EH, Hyman BT, Pietrzik CU, Berezovska O (2005). Low density lipoprotein receptor-related protein (LRP) interacts with presenilin 1 and is a competitive substrate of the amyloid precursor protein (APP) for gamma-secretase. J Biol Chem.

[B43] Dev KK, Chatterjee S, Osinde M, Stauffer D, Morgan H, Kobialko M, Dengler U, Rueeger H, Martoglio B, Rovelli G (2006). Signal peptide peptidase dependent cleavage of type II transmembrane substrates releases intracellular and extracellular signals. Eur J Pharmacol.

[B44] Casso DJ, Tanda S, Biehs B, Martoglio B, Kornberg TB (2005). Drosophila signal Peptide peptidase is an essential protease for larval development. Genetics.

[B45] Urny J, Hermans-Borgmeyer I, Schaller HC (2006). Cell-surface expression of a new splice variant of the mouse signal peptide peptidase. Biochim Biophys Acta.

[B46] Urny J, Hermans-Borgmeyer I, Gercken G, Chica Schaller H (2003). Expression of the presenilin-like signal peptide peptidase (SPP) in mouse adult brain and during development. Gene Expr Patterns.

[B47] Hope RG, McElwee MJ, McLauchlan J (2006). Efficient cleavage by signal peptide peptidase requires residues within the signal peptide between the core and E1 proteins of hepatitis C virus strain J1. J Gen Virol.

[B48] Targett-Adams P, Schaller T, Hope G, Lanford RE, Lemon SM, Martin A, McLauchlan J (2006). Signal Peptide Peptidase Cleavage of GB Virus B Core Protein Is Required for Productive Infection in Vivo. J Biol Chem.

[B49] Cupers P, Bentahir M, Craessaerts K, Orlans I, Vanderstichele H, Saftig P, De Strooper B, Annaert W (2001). The discrepancy between presenilin subcellular localization and gamma-secretase processing of amyloid precursor protein. J Cell Biol.

[B50] Majoul I, Straub M, Hell SW, Duden R, Soling HD (2001). KDEL-cargo regulates interactions between proteins involved in COPI vesicle traffic: measurements in living cells using FRET. Dev Cell.

[B51] Thinakaran G, Borchelt DR, Lee MK, Slunt HH, Spitzer L, Kim G, Ratovitsky T, Davenport F, Nordstedt C, Seeger M, Hardy J, Levey AI, Gandy SE, Jenkins NA, Copeland NG, Price DL, Sisodia SS (1996). Endoproteolysis of presenilin 1 and accumulation of processed derivatives in vivo. Neuron.

[B52] Lleo A, Berezovska O, Herl L, Raju S, Deng A, Bacskai BJ, Frosch MP, Irizarry M, Hyman BT (2004). Nonsteroidal anti-inflammatory drugs lower Abeta42 and change presenilin 1 conformation. Nat Med.

[B53] von Arnim CA, Kinoshita A, Peltan ID, Tangredi MM, Herl L, Lee BM, Spoelgen R, Hshieh TT, Ranganathan S, Battey FD, Liu CX, Bacskai BJ, Sever S, Irizarry MC, Strickland DK, Hyman BT (2005). The low density lipoprotein receptor-related protein (LRP) is a novel beta-secretase (BACE1) substrate. J Biol Chem.

[B54] Berezovska O, Lleo A, Herl LD, Frosch MP, Stern EA, Bacskai BJ, Hyman BT (2005). Familial Alzheimer's disease presenilin 1 mutations cause alterations in the conformation of presenilin and interactions with amyloid precursor protein. J Neurosci.

